# Rescue of FTLD‐associated TDP‐43 pathology and neurodegeneration by peripheral AAV‐mediated expression of brain‐penetrant progranulin

**DOI:** 10.1002/alz.090862

**Published:** 2025-01-03

**Authors:** Christian Haass

**Affiliations:** ^1^ German Center for Neurodegenerative Diseases (DZNE), Munich, Bavaria Germany

## Abstract

**Background:**

Progranulin (PGRN) haploinsufficiency is a major risk factor for frontotemporal lobar degeneration with TDP‐43 pathology (FTLD‐*GRN*). Multiple therapeutic strategies are in clinical development to restore PGRN levels in the CNS, including gene therapy. However, a limitation of current gene therapy approaches aimed to alleviate FTLD‐associated pathologies may be their inefficient brain exposure and biodistribution.

**Method:**

We therefore developed an adeno‐associated virus (AAV) targeting the liver (L) to achieve sustained peripheral expression of a transferrin receptor (TfR) binding, brain‐penetrant (b) PGRN variant (AAV(L):bPGRN) in two mouse models of FTLD‐*GRN*, namely *Grn* knockout and *GrnxTmem106b* double knockout mice. This therapeutic strategy avoids potential safety and biodistribution issues of CNS‐administered AAVs while maintaining sustained levels of PGRN in the brain following a single dose.

**Result:**

AAV(L):bPGRN treatment reduced several FTLD‐*GRN* associated disease pathologies including severe motor function deficits, aberrant TDP‐43 solubility and phosphorylation, dysfunctional protein degradation, lipid metabolism, gliosis and neurodegeneration in the brain. Translatability of our findings was confirmed in a novel human *in vitro* model using co‐cultured human induced pluripotent stem cell (hiPSC)‐derived microglia lacking PGRN and TMEM106B and wild‐type hiPSC‐derived neurons. As in mice, aberrant TDP‐43, lysosomal dysfunction and neuronal loss were ameliorated after treatment with exogenous TfR‐binding protein transport vehicle fused to PGRN (PTV:PGRN).

**Conclusion:**

Together, our studies suggest that peripherally administered brain‐penetrant PGRN replacement strategies can ameliorate FTLD‐*GRN* relevant phenotypes including TDP‐43 pathology, neurodegeneration and behavioral deficits. Our data provide preclinical proof of concept for the use of this AAV platform for treatment of FTLD‐*GRN* and potentially other CNS disorders.

